# Associations of Involuntary Smoking with Non-Suicidal Self-Injury and Suicidal Behaviors in Early Adulthood

**DOI:** 10.3390/toxics13050412

**Published:** 2025-05-21

**Authors:** Hongyang Li, Yunyun Liu, Feiyu Yuan, Jichao Li, Xiangxin Zhang, Mingyang Wu

**Affiliations:** 1 Department of Maternal and Child Health, Xiangya School of Public Health, Central South University, No.172 Tongzipo Road, Changsha 410013, China; lihongyang@csu.edu.cn (H.L.); 8304230404@csu.edu.cn (F.Y.); 8304220525@csu.edu.cn (J.L.); 8304220232@csu.edu.cn (X.Z.); 2 School of Medical Technology, Jiangsu College of Nursing, Huai’an 223001, China; liuyunyun2022@jscn.edu.cn

**Keywords:** secondhand smoke, thirdhand smoke, non-suicidal self-injury, suicidal ideation, suicide attempts

## Abstract

Background: Previous studies have demonstrated that involuntary smoking (e.g., secondhand smoke [SHS] and thirdhand smoke [THS]) is not only associated with an increased risk of several physical health problems, such as cardiovascular disease and cancer, but also impacts mental health, including depression and anxiety. However, the relationships between SHS and THS exposure and non-suicidal self-injury (NSSI), suicidal ideation (SI), and suicide attempts (SAs) remain unclear. Methods: Participants were recruited at a Chinese vocational college via voluntary online surveys conducted on campus. Self-reported SHS exposure was determined by the frequency of contact with smokers or detecting tobacco odors in living environments, while THS was assessed through regular contact with smoker-contaminated surfaces (e.g., clothing, furniture, textiles). Logistic regression analysis was performed to evaluate the associations of SHS and THS exposure with the prevalence of NSSI, SI, and SAs in never-smoking participants. Results: The study included 5716 participants (mean age = 19.3 years; females, 85.4%). The prevalence of SHS and THS exposure was 87.6% and 77.4%, with 8.8% reporting ≥15 min of SHS exposure on at least one day per week. After controlling for potential covariates, exposure to SHS (≥15 min on at least one day per week) was significantly associated with the odds of SAs (OR [95%CI] = 1.85 [1.17–2.91]). Additionally, daily THS exposure was significantly associated with increased past-year NSSI prevalence (2.35 [1.29–4.28]) compared to those without THS exposure, with similar associations observed for SI (2.11 [1.28–3.48]) and SAs (2.40 [1.23–4.69]). Conclusions: Exposure to SHS and THS was significantly associated with increased likelihood of NSSI, SI, and SAs among young adults at a Chinese vocational college. Further studies are needed to validate these associations across more diverse populations.

## 1. Introduction

Smoking has emerged as a major global public health threat, accounting for more than 8 million deaths each year [1]. Alarmingly, its harm extends beyond active smokers, as non-smokers are passively exposed to health risks through secondhand smoke (SHS) and thirdhand smoke (THS). SHS, also known as involuntary smoking or environmental tobacco smoke, refers to the mixture of smoke emitted from burning tobacco products (sidestream smoke) and exhaled by smokers (mainstream smoke) [2]. THS constitutes residual tobacco smoke contaminants that adhere to surfaces (e.g., walls, furniture, clothing) and react with environmental chemicals to form secondary pollutants, which can re-emit into the air [3]. A meta-analysis reveals that long-term SHS exposure increases lung cancer risk by 20–30% in non-smokers [4]. The hazards associated with THS have gained increased attention in recent years. The nicotine present in its residue has been observed to react with nitrous acid, yielding nitrosamines, which have been identified as potent carcinogenic agents [5]. Experimental studies have shown that THS exposure can lead to DNA damage and hepatic metabolic dysfunction [6]. Notably, sidestream smoke contains higher concentrations of benzopyrene and carbon monoxide than mainstream smoke, suggesting SHS/THS may pose greater insidious health risks than active smoking.

Previous studies have found that involuntary smoking affects both physical and mental health [1,7,8,9,10,11]. Of particular concern is the high SHS/THS exposure among adolescents and young adults, attributed to communal living, frequent indoor activities, and underdeveloped health literacy [12]. Recent studies have demonstrated that SHS exposure in youth not only increases the risk of developing cardiovascular disease but also induces neurobehavioral dysregulation [10,13]. A systematic review reported a significant dose–response relationship between SHS and risks of depression and anxiety, and even suicidal ideation (SI) [14]. Notably, suicide is the leading cause of death among 15–34-years-olds in China [15]. Suicide, a complex public health problem, covers a continuum from SI to suicide attempts (SAs) and fatal outcomes. According to the three-step theoretical model of suicide, non-suicidal self-injury (NSSI)—a pathological compensatory behavior for emotion regulation—serves as an early warning sign of suicide [16], with a lifetime prevalence of 17–23% among 18–25-year-olds [17]. Previous research links NSSI to a 7-fold increased risk of future SAs or suicide deaths [18]. Consequently, increasing numbers of studies aim to identify determinants of NSSI, SI, and SAs for early suicide prevention. Despite high SHS (i.e., 62.9%, in 2010–2018) [19] and THS exposure rates, alongside high psychological stress (i.e., 65.2%, in 2017–2023) [20] among youth, research on SHS/THS associations with NSSI, SI, and SAs remains limited. Mechanistically, tobacco residue nicotine disrupts the hypothalamic–pituitary–adrenal (HPA) axis, enhancing emotional stress responses and cortisol rhythm dysfunction [21]; polycyclic aromatic hydrocarbons, on the other hand, may impair prefrontal cortex neuroplasticity and impulse control through the oxidative stress pathway [22]. In vitro studies confirmed that THS exposure increased neuronal levels of 8-OHdG (a marker of oxidative DNA damage) and reduced glutathione peroxidase activity [6]. Therefore, it is reasonable to hypothesize that SHS and THS exposure may be associated with an increased likelihood of NSSI, SI, and SA.

Therefore, we conducted this study to examine associations between SHS and THS exposure and NSSI, SI, and SAs among college-aged youth, while accounting for potential confounders including age, sex, Body Mass Index (BMI), parental education, physical activity, academic stress, sexual orientation, sleep disturbances, and alcohol consumption [14,23,24,25,26]. College students were selected as the target population due to their unique transitional stage of psychosocial development, during which they confront distinct stressors (e.g., academic pressure, identity formation) and evidence a relatively high prevalence of mental health challenges.

## 2. Materials and Methods

### 2.1. Study Participants

This cross-sectional survey was conducted between October and November 2024 to recruit participants at a vocational college in Jiangsu, China. Jiangsu, an eastern coastal province with a developed economy and a dense population, has a strong emphasis on vocational education, particularly in healthcare-related disciplines. The overall male-to-female ratio at the study college is approximately 1:6, mainly because the nursing major has the largest enrollment. In this study, before starting the questionnaire, the structured questionnaire content and the study purpose were explained to participants so that they understood the survey’s purpose and process. First, the faculty in charge of each selected class guided students to complete the structured questionnaire via a Quick Response Code (QR Code). The online questionnaire required completion of key variables before submission to ensure no missing data. A total of 144 classes were selected to participate in this study. After excluding participants who refused to participate, 6474 individuals ultimately completed the full electronic questionnaire. Each subject provided informed consent before completing the questionnaire, and this study was conducted in accordance with the Helsinki Declaration. After excluding participants who answered quality control questions incorrectly (e.g., numerical calculation questions and similar judgment questions) (N = 157), those with repeated or missing basic information or unmatched health examination results (N = 558), and those who were active smokers or former smokers (N = 43), a total of 5716 participants were included in this study. The study protocol was reviewed and approved by the Ethics Committee of Jiangsu College of Nursing (Ethics approval Number: JSCN-ME-2024040202) on 2 April 2024.

### 2.2. Exposure Assessments

The assessment of SHS and THS refers to the Global Adult Tobacco Survey and previous studies in China [27,28,29,30,31], and we have made appropriate modifications. The Cronbach’s α value of the SHS and THS exposure assessment was 0.72 in this study. The specific questions and thresholds of SHS or THS exposure assessment are shown as follows (Table 1).

### 2.3. Outcome Assessments

The Chinese adaptation of the Ottawa Self-injury Inventory (OSI) was used to assess the presence or absence of NSSI in the past 1, 6, and 12 months. Consistent with previous research [32,33], NSSI behaviors in this study were the following behaviors that occurred in the absence of suicidal ideation: hitting, biting, picking, pulling, cutting, scalding, suffocating, dunking, swallowing, and drinking non-digestible or toxic substances. If a participant confirmed engaging in one or more of these self-injury behaviors within the specified timeframes, they were categorized as exhibiting NSSI behaviors.

Suicide ideation (SI) was assessed through the question “Have you seriously considered suicide in the past year?” Participants answering “yes” were classified as having the presence of SI. The suicide attempt (SA) metric was measured by “How many times have you attempted suicide in the past year?” Respondents reporting ≥1 attempt(s) were categorized as having a recent SA history [34].

### 2.4. Covariates

Informed by prior research and our preliminary data analysis, the analytical model incorporated demographic and behavioral covariates: age, sex, BMI, parental education, physical activity, academic stress, sexual orientation, sleep disorders, and alcohol consumption [14,23,24,25]. Parental education was operationalized as the highest educational attainment of the parent (biological, adoptive, or step-parent) who resided with the respondent for the longest duration, and was categorized into six groups: primary school or less, junior high school, high school, associate degree, bachelor’s degree or higher, and unknown/refused. Physical activity was measured by self-reported engagement in vigorous leisure-time activities during a typical week. Sleep disturbances were assessed using the Pittsburgh Sleep Quality Index (PSQI) [35]. Self-perceived academic stress was categorized into seven groups: none, low, somewhat low, moderate, somewhat high, high, or unknown/prefer not to answer. Sexual orientation in this study included heterosexual, bisexual, and homosexual. Regular alcohol consumption was defined as ≥1 drink/week for ≥6 months, based on self-reported past behavior. BMI was calculated by dividing measured body weight (kg) by the square of measured height (m).

### 2.5. Statistical Analysis

Continuous variables were presented as mean values with standard deviations (SDs) while categorical variables were presented as counts and percentages. T-tests were used for continuous variables, and χ^2^ tests were used for categorical variables. Binary logistic regression was used to analyze the relationship of SHS and THS exposure levels with the prevalence of NSSI, SI, and SAs, generating odds ratios (ORs) with 95% confidence intervals (CIs). Covariates in the logistic regression model were selected based on the published literature, including age, sex, BMI, parental education, physical activity, academic stress, sexual orientation, sleep disorders, and alcohol consumption. Statistical analysis was conducted in IBM SPSS Statistics version 27. All *p* values were from 2-sided tests, and results were deemed statistically significant at *p* < 0.05.

## 3. Results

Table 2 shows the baseline characteristics of the 5716 study participants (females, 85.4%). The mean age of all participants was 19.3 years (SD = 0.8; range = 16–25). The prevalence of ever being exposed to SHS and THS was 87.6% and 77.4% according to the frequency measurements. Among the participants, 8.8% reported being exposed to SHS for a duration exceeding 15 min on at least one day per week. Higher self-perceived academic stress, sleep disorders, physical inactivity, alcohol consumption, and non-heterosexual orientation were associated with higher prevalence of engaging in NSSI, SI, and SAs (*p* < 0.05).

Table 3 and Table 4 present the results of the regression analysis on the associations of SHS exposure with NSSI, SI, and SAs. In the crude model (Table 3), exposure to the SHS environment for more than 15 min on at least one day per week was significantly associated with increased odds of NSSI behaviors and SAs. After adjusting for potential covariates, the association between SHS exposure and the odds of NSSI became non-significant (OR_NSSI-1m_ [95%CI] = 1.47 [0.87–2.46]), whereas the association with the odds of SAs was slightly attenuated but still significant (OR [95%CI] = 1.85 [1.17–2.91]) (Table 4). In addition, exposure frequency and the number of surrounding smokers were not significantly associated with the likelihood of NSSI, SI, or SAs (Table 4).

Table 5 and Table 6 show the results of the regression analysis on the associations of THS exposure with NSSI, SI, and SAs. In the fully adjusted model (Table 6), compared to the participants who had never been exposed to THS, those with at least once a day exposure to THS had significantly increased odds of NSSI during the past year (OR [95%CI] = 2.35 [1.29–4.28]), SI (OR [95%CI] = 2.11 [1.28–3.48]), and SAs (OR [95%CI] = 2.40 [1.23–4.69]).

## 4. Discussion

### 4.1. Key Findings

This study examined the associations of involuntary smoking with NSSI and suicidal behaviors among 5716 non-smoking individuals, offering novel insights into how involuntary smoking may increase the likelihood of engaging in these behaviors. Specifically, after controlling for covariates, ≥15 min per week of SHS exposure remained a significant predictor of SAs. Additionally, THS exposure frequency was a dose-dependent risk factor for NSSI, SI, and SAs.

### 4.2. Comparison with Previous Studies

Active smoking has been shown to be associated with SI, suicide planning, and repeated SAs in prior studies, and it may increase adolescents’ vulnerability to suicidal behavior [36,37]. A growing body of research suggests that exposure to SHS also affects mental health, increasing the risk of depression, anxiety, and even attention deficit hyperactivity disorder at an early age [7,13,38,39,40,41]. Although scientists are becoming increasingly aware of the mental health risks of SHS, studies on SHS exposure and NSSI and suicidal behavior are still limited. For example, Guan et al. used data from the Global School Student Health Survey, analyzing 191,613 globally representative non-smoking adolescents, and found that SHS exposure was positively associated with SI, even after adjusting for country, region, and income level [8]. Lee et al. analyzed SHS exposure levels and mental health effects among 51,500 non-smoking Korean students, demonstrating a positive association between SHS exposure and SI after adjusting for covariates such as age, gender, and socioeconomic status [14]. In another study of Korean adolescents, Park et al. reported that SHS exposure was associated with an increased risk of SI, suicide planning, and SAs [42]. Accumulating evidence consistently demonstrates a significant positive association of SHS with mental health including suicidal behavior. Notwithstanding these insights, several limitations persist in extant studies. First, many studies employ single-dimensional exposure assessments, focusing exclusively on SHS exposure in isolated settings (e.g., schools or households), without considering cumulative exposure across multiple environments. Furthermore, some studies assessed SHS using a single question (e.g., weekly exposure days), neglecting critical factors such as exposure duration and the number of surrounding smokers, thereby failing to fully capture real-world exposure complexity.

To date, research on SHS and suicide-related behaviors has primarily focused on populations in countries like Korea, with limited studies addressing the Chinese population. The impact of SHS may vary across different countries due to differences in smoking prevalence, cultural attitudes toward smoking, and environmental factors (e.g., public smoking policies). These factors can influence both the frequency and the context of SHS exposure, potentially leading to varying mental health outcomes across different populations. The present study investigated the relationship between SHS exposure and NSSI in 5716 non-smoking individuals, providing broader evidence on the relationship between SHS exposure and suicidal behavior. Findings indicated that SHS exposure exceeding 15 min on at least one day per week was significantly associated with an increased risk of SA, though no significant correlation was observed for SHS exposure frequency. Despite the incongruity between our study and previous research, which was based on single-dimensional assessment criteria and did not account for the duration of exposure, this disparity may explain the discrepancy between the findings of our study and those of previous investigations. It suggests that duration of exposure may need to be considered when assessing the relationship between SHS exposure and suicidal behavior. In addition, while most studies consider NSSI and SI as precursors to SAs, our study found a significant association between SHS and SAs after adjusting for potential confounders, but no such association with NSSI or SI. This suggests that while NSSI and SI behaviors may also be influenced by SHS exposure, the impact of SHS on these behaviors might be more confounded by other factors, such as stress, sleep, and other individual differences. These findings underscore the complex relationship between SHS exposure and suicidal behaviors and highlight the need for further research to better understand these dynamics.

In addition to SHS, growing concern surrounds THS—residual tobacco contaminants on surfaces. Preclinical studies demonstrate THS-induced pathophysiology, including hepatic damage and insulin resistance [43]. It has been posited that exposure to THS may augment an individual’s vulnerability to gastric cancer by inducing the epithelial–mesenchymal transition pathway at the transcriptional and protein levels [44]. Short-term early exposure to THS may increase the risk of lung cancer by inducing endoplasmic reticulum stress and activating p53 signaling [45]. Furthermore, in vitro experiments have demonstrated that exposure to THS leads to an elevation in neuronal DNA oxidative damage markers (e.g., 8-OHdG). This finding suggests a potential impact on neurobehavioral systems. In recent years, a few studies have reported THS-related mental health effects [46,47], but prior work has focused on children or pregnant women (e.g., cognitive development, postnatal depression). However, there is a lack of research on the relationships of THS with NSSI and suicidal behaviors. To the best of our knowledge, the present study is the first to show evidence linking THS exposure frequency to NSSI, SI, and SAs, expanding the toxicological profile of THS beyond carcinogenesis. Notwithstanding the absence of standardized THS assessment protocols, our findings demonstrate a robust dose–response relationship (*p* < 0.001 for trend) using validated exposure frequency metrics. These novel associations warrant replication in independent cohorts employing multi-marker THS assessment (e.g., surface nicotine residues, urinary cotinine metabolites), particularly given the emerging role of THS in neuroendocrine dysregulation (HPA axis disruption) and oxidative stress pathways—biological mechanisms strongly implicated in suicidality.

### 4.3. Potential Mechanisms Underlying the Effects of SHS and THS

The mechanisms underlying the effects of SHS and THS on NSSI and suicidal behaviors remain largely unknown, but might be explained by the combination of chemical substances and environmental factors. First, hazardous substances such as nicotine, heavy metals, and volatile organic compounds [11] in SHS- and THS-exposed environments can enter the human body through the skin or by inhalation. These substances affect the neurotransmitter system and lead to anxiety and depression. Prolonged exposure to nicotine disrupts the normal functioning of the HPA axis, resulting in excessive cortisol production and altering the activity of the monoamine neurotransmitter system, which plays a key role in regulating the stress response [48]. This disruption can contribute to the development of psychological problems. Additionally, nicotine in the SHS and THS environments activates intracellular nicotinic acetylcholine receptors, which in turn increase the activity of NADPH oxidase, leading to the generation of reactive oxygen species and triggering oxidative stress. Oxidative stress leads to lipid peroxidation of cell membranes, protein oxidation, and DNA damage, thereby affecting the normal function and survival of neurons. It is also associated with reduced neuroplasticity and reduced size of brain regions, such as the prefrontal cortex and hippocampus, structural changes that are closely related to the pathogenesis of psychological disorders such as depression [49]. In terms of environmental factors, adolescents who are chronically exposed to SHS and THS may also likely experience elevated levels of psychological distress due to persistent exposure to smokers in their environment [10]. This distress exacerbates preexisting psychological issues, ultimately increasing the risk of NSSI, SI, or SAs. Since the present study does not provide results of biological mechanisms, future research focusing on the mechanisms underlying the harms of SHS and THS is highly necessary.

### 4.4. Public Health Implications

This study holds significant public health implications. First, after adjusting for covariates such as demographic variables, lifestyles, paternal factors, academic stress, and sexual orientation, the association between SHS and SAs remained significant. This finding suggests that the harmful effects of SHS on SAs may be independent of the aforementioned factors. From a population-level perspective, places where young people gather, such as universities, should not only highlight the dangers of smoking but also inform individuals about the potential harm of SHS to mental and physical health, thereby reducing exposure levels. Second, this study observed dose–response relationships between THS exposure frequency and the odds of NSSI, SI, and SA. The detrimental impact of THS remains underrecognized, particularly among younger populations, necessitating coordinated efforts from multiple stakeholders (e.g., active smokers, households with smokers, educators, public health professionals). Third, measuring THS exposure presents certain challenges, including difficulties in accurately calculating exposure duration, the hidden nature of THS exposure, and its prevalence in densely populated public spaces. Therefore, to reduce THS exposure and mitigate its public health risks, societal and governmental efforts are needed to implement policies and adopt technological measures to minimize the production and spread of THS.

### 4.5. Limitations of the Study

The present study is not without its limitations. First, the self-reported data on SHS and THS exposure were not validated through biomarker testing, which may introduce categorization bias in the exposure levels. Second, social desirability bias could have influenced self-reports, leading to underreporting or overreporting of behaviors such as SHS and THS exposure or engagement in sensitive behaviors like NSSI and suicidal ideation. The study also relied on rough approximations for covariates such as physical activity and socioeconomic status, which could affect the precision of the results. Third, although most available individual/parental demographic and lifestyle covariates were adjusted in the models, residual confounding from unmeasured parameters (e.g., housing conditions, interpersonal relationships, and physical health conditions) might influence the present findings. Finally, the study participants were first- and second-year students from a vocational college in China, primarily aged 19 to 20, with female students comprising the majority. This may limit the generalizability of the findings.

## 5. Conclusions

The present study showed that SHS was associated with the increased likelihood of SAs, whereas THS exposure was significantly associated with increased likelihood of NSSI, SI, and SAs. Further studies are needed to validate these associations across more diverse populations.

## Figures and Tables

**Table 1:** The specific questions and thresholds of SHS or THS exposure assessment.

Exposure Indicator	Dimensions	Questions	Categories	Threshold
SHS	Duration	Do you experience smoke exhaled by others for more than 15 min at least 1 day per week?	Yes; No	Yes
	Frequency	How often have you seen others with smoking behavior, or detect tobacco odors in your living/working environment?	very frequent (at least once a day), fairly frequent (5–6 times a week), usually (3–4 times a week), infrequent (1–2 times a week), very infrequent (1–3 times a month), and never	very infrequent (1–3 times a month) or more
	Number of surrounding smokers	How many smokers are present in your living/working environment?	0, 1–4, 5–9, and ≥10	≥1
THS	Frequency	How often do you come into contact with smoker-contaminated surfaces (e.g., clothing, furniture, textiles), or detect tobacco residue odors or noticeable tobacco-related flavors in indoor air?	very frequent (at least once a day), fairly frequent (5–6 times a week), usually (3–4 times a week), infrequent (1–2 times a week), very infrequent (1–3 times a month), and never	very infrequent (1–3 times a month) or more

Abbreviations: SHS: secondhand smoke; THS: thirdhand smoke.

**Table 2:** The baseline characteristics of the study participants according to non-suicidal self-injury, suicide ideation, and suicide attempts.

**Variables**	**Total**	**NSSI-1m**		***p***	**Suicide Ideation**		***p***	**Suicide Attempt**		***p***
		**No**	**Yes**		**No**	**Yes**		**No**	**Yes**	
Age, mean (SD), y	19.3 (0.8)	19.3 (0.8)	19.4 (0.9)	0.081 ^a^	19.3 (0.8)	19.3 (0.8)	0.344 ^a^	19.3 (0.8)	19.2 (0.9)	0.185 ^a^
BMI, mean (SD), kg/m^2^	22.4 (4.3)	22.4 (4.3)	22.3 (4.6)	0.494 ^a^	22.4 (4.2)	22.8 (4.8)	0.335 ^a^	22.4 (4.3)	22.5 (4.5)	0.766 ^a^
Sex, n (%)				0.156 ^b^			0.042 ^b^			0.031 ^b^
Male	836 (14.6)	810 (96.9)	26 (3.1)		779 (93.2)	57 (6.8)		801 (95.8)	35 (4.2)	
Female	4880 (85.4)	4768 (97.7)	112 (2.3)		4631 (94.9)	249 (5.1)		4743 (97.2)	137 (2.8)	
Paternal educational attainment, n (%)				0.205 ^b^			0.883 ^b^			0.149 ^b^
Primary school and below	523 (9.1)	506 (96.7)	17 (3.3)		491 (93.9)	32 (6.1)		504 (96.4)	19 (3.6)	
Junior high school	2270 (39.7)	2218 (97.7)	52 (2.3)		2150 (94.7)	120 (5.3)		2213 (97.5)	57 (2.5)	
High school	1545 (27.0)	1516 (98.1)	29 (1.9)		1466 (94.9)	79 (5.1)		1504 (97.3)	41 (2.7)	
Associate degree	579 (10.1)	561 (96.9)	18 (3.1)		548 (94.6)	31 (5.4)		556 (96.0)	23 (4.0)	
Bachelor’s degree and above	439 (7.7)	430 (97.9)	9 (2.1)		415 (94.5)	24 (5.5)		416 (94.8)	23 (5.2)	
Do not know/Refuse to answer	360 (6.3)	347 (96.4)	13 (3.6)		340 (94.4)	20 (5.6)		351 (97.5)	9 (2.5)	
Maternal educational attainment, n (%)				0.010 ^b^			0.826 ^b^			0.709 ^b^
Primary school and below	994 (17.4)	963 (96.9)	31 (3.1)		928 (93.4)	66 (6.6)		950 (95.6)	44 (4.4)	
Junior high school	2315 (40.5)	2266 (97.9)	49 (2.1)		2206 (95.3)	109 (4.7)		2261 (97.7)	54 (2.3)	
High school	1202 (21.0)	1186 (98.7)	16 (1.3)		1135 (94.4)	67 (5.6)		1177 (97.9)	25 (2.1)	
Associate degree	497 (8.7)	480 (98.6)	17 (3.4)		472 (95.0)	25 (5.0)		476 (95.8)	21 (4.2)	
Bachelor’s degree and above	333 (5.8)	320 (96.1)	13 (3.9)		319 (95.8)	14 (4.2)		316 (94.9)	17 (5.1)	
Do not know/Refuse to answer	375 (6.6)	363 (96.8)	12 (3.2)		350 (93.3)	25 (6.7)		364 (97.1)	11 (2.9)	
Academic pressure, n (%)				<0.001 ^b^			<0.001 ^b^			<0.001 ^b^
None	330 (5.8)	321 (97.3)	9 (2.7)		318 (96.4)	12 (3.6)		317 (96.1)	13 (3.9)	
Low	252 (4.4)	247 (98.0)	5 (2.0)		231 (91.7)	21 (8.3)		244 (96.8)	8 (3.2)	
Somewhat low	407 (7.1)	4019 (98.5)	6 (1.5)		386 (94.8)	21 (5.2)		402 (98.8)	5 (1.2)	
Moderate	3504 (61.3)	3439 (98.1)	65 (1.9)		3357 (95.8)	147 (4.2)		3426 (97.8)	78 (2.2)	
Somewhat high	744 (13.0)	720 (96.8)	24 (3.2)		697 (93.7)	47 (6.3)		714 (96.0)	20 (4.0)	
High	174 (3.0)	166 (95.4)	8 (4.6)		151 (86.8)	23 (13.2)		163 (93.7)	11 (6.3)	
Don’t know/Prefer not to say	305 (5.3)	284 (93.1)	21 (6.9)		270 (88.5)	35 (11.5)		278 (91.1)	27 (8.9)	
Sexual orientation, n (%)				<0.001 ^b^			<0.001 ^b^			<0.001 ^b^
Heterosexuality	5371 (94.0)	5254 (97.8)	117 (2.2)		5113 (95.2)	258 (4.8)		5235 (97.5)	136 (2.5)	
Homosexuality	244 (4.3)	228 (93.4)	16 (6.5)		211 (86.5)	33 (13.5)		220 (90.2)	24 (9.8)	
Bisexuality	101 (1.8)	96 (95.0)	5 (5.0)		86 (85.1)	15 (14.9)		89 (88.1)	12 (11.9)	
Alcohol consumption, n (%)				0.028 ^b^			0.005 ^b^			0.014 ^b^
No	5657 (99.0)	5523 (97.6)	134 (2.4)		5359 (94.7)	298 (5.3)		5490 (97.0)	167 (3.0)	
Yes	59 (1.0)	55 (93.2)	4 (6.8)		51 (86.4)	8 (13.6)		54 (91.5)	5 (8.5)	
Sleep disorders, n (%)				<0.001 ^b^			<0.001 ^b^			<0.001 ^b^
No	4813 (84.2)	4742 (98.5)	71 (1.5)		4626 (96.1)	187 (3.9)		4716 (98.0)	97 (2.0)	
Yes	903 (15.8)	836 (92.6)	67 (7.4)		784 (86.8)	119 (13.2)		828 (91.7)	75 (8.3)	
Physical activity, n (%)				0.144 ^b^			0.009 ^b^			0.028 ^b^
No	3425 (59.9)	3334 (97.3)	91 (2.7)		3220 (94.0)	205 (6.0)		3308 (96.6)	117 (3.4)	
Yes	2291 (40.1)	2244 (97.9)	47 (2.1)		2190 (95.6)	101 (4.4)		2236 (97.6)	55 (2.4)	
SHS exposure ≥ 15 min on ≥1 day/week, n (%)				<0.001 ^b^			<0.001 ^b^			<0.001 ^b^
NO	5213 (91.2)	5100 (97.8)	113 (2.2)		4951 (95.0)	262 (5.0)		5076 (97.4)	137 (2.6)	
YES	503 (8.8)	478 (95.0)	25 (5.0)		459 (91.3)	44 (8.7)		468 (93.0)	35 (7.0)	
Frequency of SHS exposure, n (%)				0.002 ^b^			<0.001 ^b^			<0.001 ^b^
Never	706 (12.4)	696 (98.6)	10 (1.4)		668 (94.6)	38 (5.4)		686 (97.2)	20 (2.8)	
Very infrequent	1530 (26.8)	1500 (18.0)	30 (2.0)		1465 (95.8)	65 (4.2)		1499 (98.0)	31 (2.0)	
Infrequent	1321 (23.1)	1285 (97.3)	36 (2.7)		1253 (94.9)	68 (5.1)		1286 (97.4)	35 (2.6)	
Moderate	1269 (22.2)	1242 (97.9)	27 (2.1)		1207 (95.1)	62 (4.9)		1233 (97.2)	36 (2.8)	
Fairly frequent	501 (8.8)	481 (96.0)	20 (4.0)		470 (93.8)	31 (6.2)		472 (94.2)	29 (5.8)	
Very frequent	389 (6.8)	374 (96.1)	15 (3.9)		347 (89.2)	42 (10.8)		368 (94.6)	21 (5.4)	
Number of surrounding smokers, n (%)				<0.001 ^b^			0.003 ^b^			0.003 ^b^
0	3294 (57.6)	3231 (98.1)	63 (1.9)		3144 (95.4)	150 (4.6)		3211 (97.5)	83 (2.5)	
1–4	1787 (31.3)	1740 (97.4)	47 (2.6)		1676 (93.8)	111 (6.2)		1726 (96.6)	61 (3.4)	
5–9	324 (5.7)	311 (96.0)	13 (4.0)		300 (92.6)	24 (7.4)		312 (96.3)	12 (3.7)	
≥10	311 (5.4)	296 (95.2)	15 (4.8)		290 (93.2)	21 (6.8)		295 (94.9)	16 (5.1)	
Frequency of THS exposure, n (%)				<0.001 ^b^			<0.001 ^b^			<0.001 ^b^
Never	1291 (22.6)	1272 (98.5)	19 (1.5)		1229 (95.2)	62 (4.8)		1262 (97.8)	29 (2.2)	
Very infrequent	1793 (31.4)	1761 (98.2)	32 (1.8)		1716 (95.7)	77 (4.3)		1751 (97.7)	42 (2.3)	
Infrequent	1152 (20.2)	1116 (96.9)	36 (3.1)		1080 (93.8)	72 (6.3)		1121 (97.3)	31 (2.7)	
Moderate	971 (17.0)	942 (97.0)	29 (3.0)		929 (95.7)	42 (4.3)		936 (96.4)	35 (3.6)	
Fairly frequent	303 (5.3)	292 (96.4)	11 (3.6)		279 (92.1)	24 (7.9)		285 (94.1)	18 (5.9)	
Very frequent	206 (3.6)	195 (94.7)	11 (5.3)		177 (85.9)	29 (14.1)		189 (91.7)	17 (8.3)	

Abbreviations: BMI: Body Mass Index; SHS: secondhand smoke; THS: thirdhand smoke; NSSI: non-suicidal self-injury. ^a^, *p* value from *t*-test; ^b^, *p* value from chi-square test.

**Table 3:** The association of secondhand smoke exposure and the prevalence of non-suicidal self-injury, suicide ideation, and suicide attempts in crude model.

SHS Exposure	NSSI-1m [OR (95%CI)]	NSSI-6m [OR (95%CI)]	NSSI-12m [OR (95%CI)]	Suicide Ideation [OR (95%CI)]	Suicide Attempt [OR (95%CI)]
SHS exposure ≥ 15 min on ≥1 day/week					
No	Ref	Ref	Ref	Ref	Ref
Yes	1.84 (1.12–3.00)	1.56 (1.02–2.38)	1.64 (1.13–2.38)	1.38 (0.96–1.99)	2.12 (1.38–3.24)
Frequency of SHS exposure					
Never	Ref	Ref	Ref	Ref	Ref
Very infrequent	1.35 (0.65–2.80)	1.24 (0.70–2.18)	1.03 (0.64–1.67)	0.74 (0.49–1.12)	0.70 (0.40–1.25)
Infrequent	1.74 (0.84–3.58)	1.53 (0.87–2.70)	1.22 (0.75–1.99)	0.85 (0.56–1.30)	0.88 (0.49–1.55)
Moderate	1.21 (0.56–2.59)	1.14 (0.62–2.07)	0.98 (0.59–1.63)	0.76 (0.49–1.18)	0.87 (0.48–1.56)
Fairly frequent	2.03 (0.89–4.60)	1.90 (0.99–3.65)	1.55 (0.88–2.71)	0.91 (0.54–1.54)	1.65 (0.87–3.12)
Very frequent	1.70 (0.70–4.11)	1.59 (0.78–3.23)	1.36 (0.74–2.50)	1.59 (0.96–2.65)	1.31 (0.66–2.64)
*p* for trend	0.002	0.001	<0.001	<0.001	<0.001
Number of surrounding smokers					
0	Ref	Ref	Ref	Ref	Ref
1–4	1.17 (0.78–1.76)	1.23 (0.88–1.70)	1.34 (1.00–1.80)	1.29 (0.98–1.70)	1.11 (0.76–1.60)
5–9	1.72 (0.90–3.27)	1.21 (0.67–2.19)	1.57 (0.96–2.56)	1.49 (0.93–2.38)	1.07 (0.56–2.04)
≥10	1.88 (1.00–3.54)	1.70 (0.99–2.91)	1.56 (0.95–2.58)	1.18 (0.71–1.97)	1.29 (0.70–2.35)

Abbreviations: SHS: secondhand smoke; NSSI: non-suicidal self-injury; OR: odds ratio; CI: confidence interval. These results were derived from logistic regression models.

**Table 4:** The association of secondhand smoke exposure and the prevalence of non-suicidal self-injury, suicide ideation, and suicide attempts in the fully adjusted model.

SHS Exposure	NSSI-1m [OR (95%CI)]	NSSI-6m [OR (95%CI)]	NSSI-12m [OR (95%CI)]	Suicide Ideation [OR (95%CI)]	Suicide Attempt [OR (95%CI)]
SHS Exposure ≥ 15 min on ≥1 day/week					
No	Ref	Ref	Ref	Ref	Ref
Yes	1.47 (0.87–2.46)	1.34 (0.86–2.11)	1.48 (1.00–2.20)	1.21 (0.82–1.78)	1.85 (1.17–2.91)
Frequency of SHS exposure					
Never	Ref	Ref	Ref	Ref	Ref
Very infrequent	1.39 (0.66–2.91)	1.22 (0.68–2.18)	1.00 (0.61–1.64)	0.71 (0.46–1.08)	0.77 (0.42–1.39)
Infrequent	1.70 (0.81–3.56)	1.49 (0.83–2.68)	1.17 (0.71–1.93)	0.84 (0.54–1.29)	0.96 (0.53–1.74)
Moderate	1.15 (0.53–2.50)	1.04 (0.56–1.93)	0.88 (0.52–1.48)	0.72 (0.46–1.13)	0.96 (0.52–1.75)
Fairly frequent	1.85 (0.80–4.24)	1.68 (0.86–3.28)	1.36 (0.77–2.43)	0.80 (0.47–1.37)	1.68 (0.88–3.24)
Very frequent	1.15 (0.46–2.85)	1.05 (0.50–2.20)	0.94 (0.50–1.78)	1.19 (0.70–2.02)	0.93 (0.44–1.94)
*p* for trend	0.136	0.183	0.091	0.073	0.018
Number of surrounding smokers					
0	Ref	Ref	Ref	Ref	Ref
1–4	1.21 (0.80–1.83)	1.24 (0.88–1.73)	1.35 (1.00–1.82)	1.30 (0.98–1.72)	1.15 (0.79–1.67)
5–9	1.56 (0.80–3.02)	1.04 (0.56–1.94)	1.37 (0.82–2.29)	1.40 (0.86–2.28)	0.96 (0.49–1.88)
≥10	1.58 (0.82–3.07)	1.40 (0.79–2.48)	1.29 (0.76–2.19)	1.00 (0.58–1.72)	0.98 (0.51–1.88)

Abbreviations: SHS: secondhand smoke; NSSI: non-suicidal self-injury; OR: odds ratio; CI: confidence interval. These results were derived from logistic regression models, adjusted for covariates including age, sex, BMI, father’s education, mother’s education, physical activity, academic pressure, sexual orientation, alcohol consumption, and sleep disorders.

**Table 5:** The association of thirdhand smoke exposure and the prevalence of non-suicidal self-injury, suicide ideation, and suicide attempts in crude model.

Frequency of THS Exposure	NSSI-1m [OR (95%CI)]	NSSI-6m [OR (95%CI)]	NSSI-12m [OR (95%CI)]	Suicide Ideation [OR (95%CI)]	Suicide Attempt [OR (95%CI)]
Never	Ref	Ref	Ref	Ref	Ref
Very infrequent	1.22 (0.69–2.16)	1.43 (0.89–2.28)	1.42 (0.95–2.12)	0.89 (0.63–1.25)	1.04 (0.65–1.68)
Infrequent	2.16 (1.23–3.79)	2.53 (1.59–4.01)	2.06 (1.37–3.11)	1.32 (0.93–1.87)	1.20 (0.72–2.01)
Moderate	2.06 (1.15–3.70)	1.91 (1.16–3.14)	1.84 (1.19–2.84)	0.90 (0.60–1.34)	1.63 (0.99–2.68)
Fairly frequent	2.52 (1.19–5.36)	2.78 (1.50–5.17)	2.92 (1.72–4.95)	1.70 (1.05–2.78)	2.75 (1.50–5.02)
Very frequent	3.78 (1.77–8.06)	3.41 (1.76–6.62)	3.64 (2.07–6.41)	3.25 (2.03–5.19)	3.91 (2.11–7.26)
*p* for trend	<0.001	<0.001	<0.001	<0.001	<0.001

Abbreviations: THS: thirdhand smoke; NSSI: non-suicidal self-injury; OR: odds ratio; CI: confidence interval. These results were derived from logistic regression models.

**Table 6:** The association of thirdhand smoke exposure and the prevalence of non-suicidal self-injury, suicide ideation, and suicide attempts in the fully adjusted model.

Frequency of THS Exposure	NSSI-1m [OR (95%CI)]	NSSI-6m [OR (95%CI)]	NSSI-12m [OR (95%CI)]	Suicide Ideation [OR (95%CI)]	Suicide Attempt [OR (95%CI)]
Never	Ref	Ref	Ref	Ref	Ref
Very infrequent	1.18 (0.66–2.12)	1.39 (0.86–2.25)	1.37 (0.91–2.08)	0.83 (0.59–1.18)	1.12 (0.68–1.83)
Infrequent	1.92 (1.08–3.41)	2.27 (1.41–3.66)	1.84 (1.20–2.80)	1.18 (0.82–1.69)	1.14 (0.67–1.94)
Moderate	1.69 (0.92–3.08)	1.64 (0.98–2.75)	1.59 (1.02–2.49)	0.77 (0.51–1.16)	1.56 (0.93–2.62)
Fairly frequent	1.83 (0.84–3.98)	2.04 (1.07–3.90)	2.22 (1.27–3.85)	1.30 (0.78–2.17)	2.26 (1.20–4.26)
Very frequent	2.19 (0.99–4.83)	2.02 (1.00–4.06)	2.35 (1.29–4.28)	2.11 (1.28–3.48)	2.40 (1.23–4.69)
*p* for trend	0.012	0.009	<0.001	0.028	<0.001

Abbreviations: THS: thirdhand smoke; NSSI: non-suicidal self-injury; OR: odds ratio; CI: confidence interval. These results were derived from logistic regression models, adjusted for covariates including age, sex, BMI, father’s education, mother’s education, physical activity, academic pressure, sexual orientation, alcohol consumption, and sleep disorders.

## Data Availability

The data set will be available from the corresponding author, upon reasonable request.

## Abbreviations

The following abbreviations are used in this manuscript:

SHS	secondhand smoke
THS	thirdhand smoke
NSSI	non-suicidal self-injury
SA	suicide attempt
SI	suicidal ideation
BMI	Body Mass Index
